# Critical Early Roles for *col27a1a* and *col27a1b* in Zebrafish Notochord Morphogenesis, Vertebral Mineralization and Post-embryonic Axial Growth

**DOI:** 10.1371/journal.pone.0008481

**Published:** 2009-12-29

**Authors:** Helena E. Christiansen, Michael R. Lang, James M. Pace, David M. Parichy

**Affiliations:** 1 Molecular and Cellular Biology Program, University of Washington, Seattle, Washington, United States of America; 2 Department of Biology, University of Washington, Seattle, Washington, United States of America; 3 Department of Pathology, University of Washington, Seattle, Washington, United States of America; Harvard University, United States of America

## Abstract

**Background:**

Fibrillar collagens are well known for their links to human diseases, with which all have been associated except for the two most recently identified fibrillar collagens, type XXIV collagen and type XXVII collagen. To assess functions and potential disease phenotypes of type XXVII collagen, we examined its roles in zebrafish embryonic and post-embryonic development.

**Methodology/Principal Findings:**

We identified two type XXVII collagen genes in zebrafish, *col27a1a* and *col27a1b*. Both *col27a1a* and *col27a1b* were expressed in notochord and cartilage in the embryo and early larva. To determine sites of type XXVII collagen function, *col27a1a* and *col27a1b* were knocked down using morpholino antisense oligonucleotides. Knockdown of *col27a1a* singly or in conjunction with *col27a1b* resulted in curvature of the notochord at early stages and formation of scoliotic curves as well as dysmorphic vertebrae at later stages. These defects were accompanied by abnormal distributions of cells and protein localization in the notochord, as visualized by transmission electron microscopy, as well as delayed vertebral mineralization as detected histologically.

**Conclusions/Significance:**

Together, our findings indicate a key role for type XXVII collagen in notochord morphogenesis and axial skeletogenesis and suggest a possible human disease phenotype.

## Introduction

Spinal deformity is a common condition that can profoundly reduce the quality of life for affected individuals. Scoliosis, in particular, is highly prevalent in the human population, with prevalence estimates ranging from 1.4% to 20%, depending on the ages surveyed [1]–[3]. The many forms of scoliosis are generally distinguished by age of onset. Congenital scoliosis is usually caused by abnormal vertebral development *in utero*. In adolescent idiopathic scoliosis (AIS), vertebrae are formed normally and spinal deformity begins during adolescence, probably reflecting abnormalities in muscle and ligament growth. Finally, degenerative scoliosis is found in aging adults and can be caused by changes in vertebral body structure due to fracture, as well as alterations in surrounding musculature and ligaments [4].

The human vertebral column comprises mineralized vertebrae each separated by an unmineralized cushion called the intervertebral disc. The vertebral column is derived from the notochord, a stiff rod-shaped structure that serves as an axial support for the developing embryo. In mammals, a portion of the notochord is maintained throughout life in the center of the intervertebral discs, the nucleus pulposus. Two of the main components of the vertebrae and intervertebral discs are collagens and proteoglycans. Collagens provide tensile strength, serve as a scaffold for other extracellular matrix molecules such as proteoglycans, and provide a template for mineral deposition. Proteoglycans hold water and thus provide resistance to compressive forces [5]–[6].

Collagens are extracellular matrix proteins characterized by domains consisting of Gly-Xaa-Yaa repeats in which glycine is in every third position and Xaa and Yaa can be a variety of amino acids but, most commonly, are proline and 4-hydroxyproline, respectively. There are five classes of collagens, encoded by more than 42 genes that generate the chains of some 28 types of collagens in five major classes [7]. The fibril-forming collagens (types I, II, III, V, XI, XXIV and XXVII) have been studied most extensively due to their abundance and links to human disease. With the exception of the most recently identified types XXIV and XXVII, mutations in fibrillar collagen genes have been shown to give rise to genetic disorders. Mutations in these genes are responsible for osteogenesis imperfecta (OI), also known as brittle bone disease, forms of chondrodysplasias and forms of Ehlers-Danlos syndrome [7].

Types XXIV and XXVII collagen, which cluster into a separate evolutionary clade, share characteristics that distinguish them from the classical fibrillar collagens [8]–[9]. These features include a globular N-propeptide domain lacking a minor triple helix and a triple helical domain of 997 amino acids that is shorter than the 1014–1029 amino acids for classical fibrillar collagens. The helical domain also contains two short interruptions of the Gly-Xaa-Yaa repeats. Since interruptions in the helical domain of the classical fibrillar collagens are disease-causing, this suggests that types XXIV and XXVII collagen function in a manner distinct from that of the classical fibrillar collagens. No function has been described for either collagen belonging to this clade.

In this study, we identified two genes for type XXVII collagen in zebrafish, *col27a1a* and *col27a1b*, corresponding to a single gene in humans. Both *col27a1a* and *col27a1b* are expressed in the notochord and cartilage elements. Embryos deficient in *col27a1a* have delayed vertebral mineralization, form dysmorphic vertebrae, and develop scoliotic vertebral curves. Simultaneous knockdown with individually sub-effective doses of *col27a1a* and *col27a1b* also results in formation of dysmorphic vertebrae, delayed mineralization, and the development of scoliosis, indicating that they both play important roles in notochord and vertebral development. Our results indicate a key role for type XXVII collagen function in the notochord and suggest that human disease phenotypes that include significant scoliosis are candidates for mutations in *COL27A1*.

## Materials and Methods

### Fish Stocks

Zebrafish *Danio rerio* were from in-crosses of a wild-type stock, WT (WA), which is itself derived each generation by intercrossing the inbred mapping strains AB^wp^ and wik. Fish were maintained at 28°C on a 14∶10 hour light∶dark cycle. Staging for embryos followed Kimmel et al. [10]. Staging for post-embryonic larvae followed Parichy et al. [11], and employed standardized standard length (SSL) measurements that account for variation in development rate and stage across fish stocks and laboratories. Also provided are ages post-fertilization, though these are not themselves sufficient indicators of developmental progress at post-embryonic stages [11]. Experiments were done in accordance with approved University of Washington IACUC protocols.

### Identification of Zebrafish *col27a1a* and *col27a1b*

Two type XXVII collagen genes were identified on chromosomes 21 and 5 of the zebrafish genome, *col27a1a* and *col27a1b*, respectively. Genescan predictions from the Ensemble genome browser were used to identify two putative *col27a1* loci followed by computational analysis to obtain the full triple helical and C-propeptide regions of *col27a1a* and the complete *col27a1b* sequence.

### PCR, 5′ RACE and Sequencing

Total RNA was isolated from zebrafish embryos with Trizol (Invitrogen) according to manufacturer's instructions. Complementary DNA was synthesized using Superscript II reverse transcriptase (Invitrogen) with oligo-dT priming and computationally predicted transcripts were confirmed by sequencing. To obtain a complete cDNA for *col27a1a*, we performed 5′ RACE using the First Choice RLM-RACE Kit (Ambion).

### Phylogenetic Analysis

Fibrillar collagen sequences from human, mouse, zebrafish, chicken, and sea squirt were aligned with CLUSTALX2.0, and the conserved region of the C-propeptide was manually selected. MrBayes version 3.0 was used to generate Bayesian inference topologies [12]. Analyses were run for 10^6^ generations with tree samples every 100 generations by which point the average standard deviation of split frequencies had dropped below 0.1. We plotted likelihood values against generation number to determine when likelihood values had stabilized. The first 4400 trees were discarded as ‘burn-in’ before the values stabilized, and a consensus tree was generated with Bayesian posterior probability values. A full heuristic parsimony analysis with 1000 bootstrap replicates was also performed using PAUP*4.0b10 (data not shown). The tree generated from this analysis was identical to the Bayesian tree with the exception that in the full heuristic parsimony analysis, the *col27a1* clade was basal to all other collagens of the in-group. The following GenBank sequences were used: *Homo sapiens COL1A1* NP_000079, *COL1A2* NP_000080, *COL2A1* NP_001835, *COL3A1* NP_000081, *COL5A1* NP_000084, *COL5A2* NP_000384, *COL5A3* NP_056534, *COL11A1* NP_001845, *COL11A2* NP_542411, *COL24A1* NP_690850, *COL27A1* NP_116277; *Mus musculus Col1a1* NP_031768, *Col1a2* NP_031769, *Col2a1* NP_112440, *Col3a1* NP_034060, *Col5a1* NP_056549, *Col5a2* NP_031763, *Col5a3* NP_058615, *Col11a1* NP_031755, *Col11a2* NP_034056, *Col24a1* NP_082046, *Col27a1* NP_079961; *Danio rerio col1a1* NP_954684, *col1a2* NP_892013, *col1a3* NP_958886, *col2a1a* NP_571367, *col11a1* NP_001073461, *col27a1a* GQ229459, *col27a1b* EF032484; *Gallus gallus col1a1* P02457, *col1a2* P02467, *col2a1* NP_989757, *col3a1* P12105, *col5a1* NP_990121, *col5a2* XP_421846, *col11a1* XP_001231624, *col24a1* XP_422363; *Ciona intestinalis CiFCol2* AK113159. Since sequence data was unavailable for *Danio rerio col5a2* and *Gallus gallus col27a1*, protein sequence predictions, ENSDARP00000041806 and ENSGALP00000011258, respectively, were used.

### *In Situ* Hybridization

Standard protocols were used for analyses of *col27a1a* and *col27a1b* mRNA tissue distribution in embryos [13]. Wild-type embryos collected at 2–5 days-post-fertilization (dpf) were treated with 1-phenyl-2-thiourea (PTU) over the course of development to prevent melanization [14].

### Morpholino Injection

One- or two-cell staged embryos were injected with antisense morpholino oligonucleotides (Gene Tools, Philomath, OR) at a volume of 1-2 nl at the following dosages: *col27a1a* SBMO 5-20ng; *col27a1a* SBmmMO 5-20ng; *col27a1a* TBMO 1ng; *col27a1a* TBmmMO 1ng; *col27a1b* SBMO 3–15 ng; *col27a1b* TBMO 1–10 ng; *col27a1b* TBmmMO 1–3 ng. Morpholino sequences were as follows (start codons are underlined and mispaired nucleotides are in lowercase):

*col27a1a SBMO* [5′-CAGCACATACGCACCATCGTAAAGC-3′]

*col27a1a SBmmMO* [5′-CAcCAgATACGgACCATCcTAAAcC-3′]

*col27a1a TBMO* [5′-TCCTTCGGGTCGCTAAATTCATTTG-3′]

*col27a1a TBmmMO* [5′-TCgTTCaGGTCGgTAAATTgATaTG-3′]

*col27a1b SBMO* [5′-CAATGCAGTTTACAATCTCACCATC-3′]

*col27a1b TBMO* [5′-TATTGTCCGGCTCCATCGCGCTTAC-3′]

*col27a1b TBmmMO* [5′-TATTcTCCcGCTgCATCcCcCTTAC-3′]

Stocks were diluted to 0.8–1.0 mM in water and final dilutions were made in Danieau buffer (58 mM NaCl, 0.7 mM KCl, 0.4 mM MgSO_4_, 0.6 mM Ca(NO_3_)_2_, 5 mM Hepes, pH 7.6) or H_2_O. Abnormal splicing for splice-blocking morpholinos was confirmed by RT-PCR.

### Histology

Juvenile and adult fish were fixed in 4% paraformaldehyde in PBS for 48 hr at 4°C and dehydrated to 100% ethanol over 4 days. They were stained with Alcian blue and Alizarin red following a standard protocol for staining of larval fish [15]. Fish were transferred to 100% glycerol over 1 week before analysis and storage.

Calcein staining was performed as described with minor modifications [16]. Briefly, fish were anesthetized in 0.6 mM MS222 (Sigma) buffered to pH 7.0 and immersed in 0.2% calcein in 10% Hanks solution buffered to pH 7.2 for 10 min followed by three 10 min washes in Hanks solution. Larvae were scored for the number of vertebrae that had begun to mineralize. Any vertebra that had begun to mineralize was counted.

Fish were examined using Olympus SZX-12 or Zeiss Lumar stereomicroscopes, or with Zeiss Axioplan 2 or Zeiss Observer compound microscopes. Digital images were collected with Zeiss Axiocam HR cameras using Zeiss Axiovision 3.

### Transmission Electron Microscopy

Embryos were fixed in one-half Karnovsky's fixative overnight, postfixed with 1.0% osmium tetroxide and stained en bloc with uranyl acetate in cacodylate buffer. Tissues were dehydrated through a graded ethanol series, and embedded in Epon. Semi-thin sections (300 nm) were cut using a Leica Ultracut UCT ultramicrotome and stained with Richardson's stain for 30 seconds to visualize morphology. Ultrathin sections (70 nm) were cut as above, transferred onto formvar-coated copper grids and stained with lead citrate, uranyl acetate and 1% phosphotungstic acid. Samples were analyzed using a Jeol 1200 TEM.

### Statistical Analysis

Vertebral ossification was analyzed by parametric analyses of variance after verifying that residuals were normally distributed with equal variances across treatments. We used morpholino treatment as a fixed effect and we included clutch as a factor to control for minor but statistically significant variation among sibships. We present below least squares means that control for inter-clutch variability. We did not find significant clutch x treatment interactions indicating that families did not respond significantly differently to morpholino knockdowns (data not shown). To test if effects of morpholino knockdown on vertebral ossification could be explained by developmental delays alone, we used analyses of covariance including individual standard length, mineralized vertebral number, and morpholino treatment. All statistical analyses were performed with JMP 8.0.1 (SAS Institute, Cary NC) for Macintosh.

## Results

### Identification of Type XXVII Collagen Genes in Zebrafish

**Figure 1:**
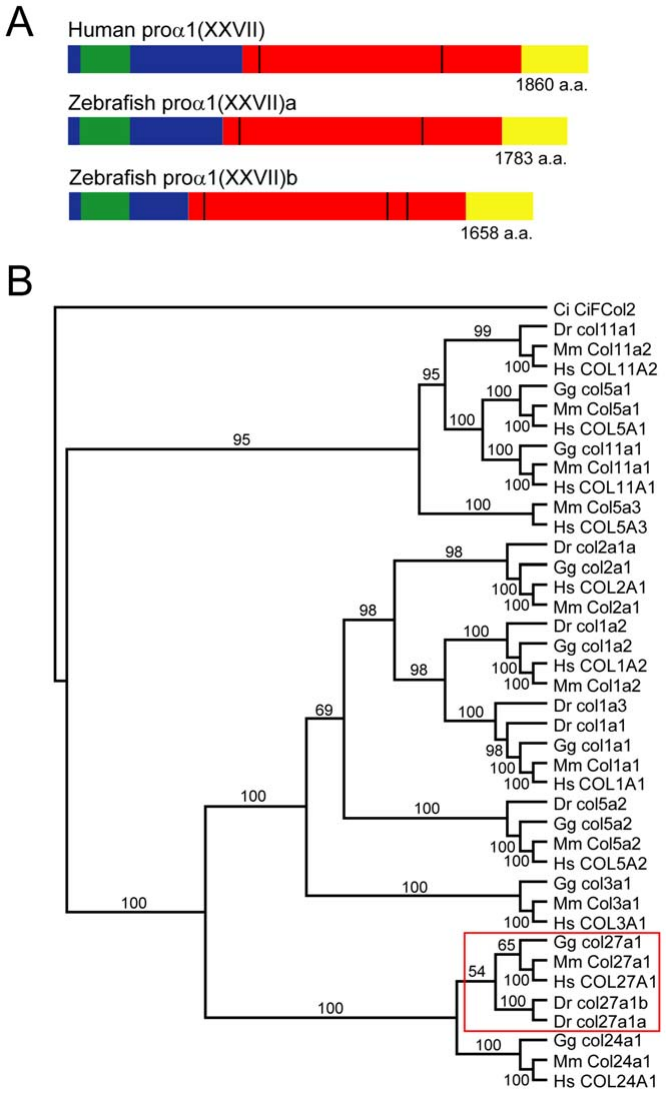
Identification of two type XXVII collagen genes in zebrafish. (A) Protein schematic showing domains of human and zebrafish type XXVII collagen. Blue: N-propeptide, Green: thrombospondin domain, Red: triple helical domain, Yellow: C-propeptide, Black: interruptions of the triple helix. Total protein length is listed in amino acids. (B) Fibrillar collagen phylogeny based on amino acid sequences from the conserved C-propeptide. Bayesian tree with posterior probabilities is shown. The two predicted type XXVII collagen proteins in zebrafish cluster with those of chicken, mouse and human (red box). Ci, *Ciona intestinalis*; Dr, *Danio rerio*; Gg, *Gallus gallus*; Mm, *Mus musculus*; Hs, *Homo sapiens*.

Two type XXVII collagen genes were identified on chromosomes 21 and 5 of the zebrafish genome, *col27a1a* (GenBank accession no. GQ229459) and *col27a1b* (GenBank accession no. EF032484), respectively (Fig. 1A). Predicted proteins for the zebrafish and human type XXVII collagen genes indicated that both zebrafish proteins contained a conserved thrombospondin domain and shared the same exon structure with the human protein. Both forms of the zebrafish type XXVII collagen contained the two interruptions of the triple helix found in the human protein. However, proα(XXVII)b contained an additional interruption in the triple helix.

To verify the orthology of zebrafish *col27a1* genes with those of mammals, we performed a Bayesian inference phylogenetic analysis of fibrillar collagens using amino acid sequences from the conserved C-terminus propeptides. This analysis showed that both *col27a1a* and *col27a1b* clustered with *COL27A1* and *Col27a1* from human and mouse, respectively, thereby, confirming the orthology of zebrafish and mammalian genes and showing that the zebrafish genes are paralogues of one another (Fig. 1B).

### Expression of *col27a1a* and *col27a1b* in Notochord and Cartilage

**Figure 2:**
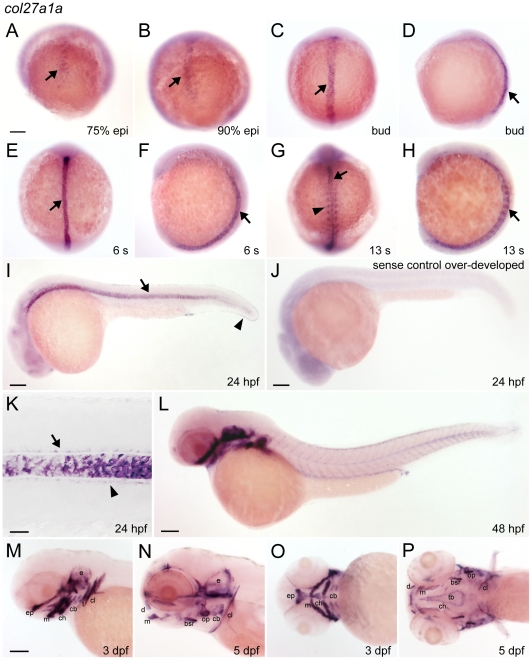
*col27a1a* is expressed in notochord and cartilage. (A–I) *col27a1a* was expressed in the notochord at early stages (black arrow), and (G) in the adaxial cells of the somites by 13 somite stage (arrowhead). At 24 hpf, expression was also observed in the (I) fin fold (black arrowhead) and in the (K) floor plate (black arrow) and hypochord (black arrowhead), surrounding the notochord. (J) Sense probe control was intentionally overexposed and revealed an absence of notochord staining. (L–P) Beginning at 48 hpf, strong expression of *col27a1a* was seen in the cartilage elements of the head and continued through 5 dpf, the latest time point analyzed. (M) Expression was also seen in the first forming tooth. Scale bar in K represents 20 µm. Scale bars in remaining images represent 100 µm. Scale bar in A applies to B–H. Scale bar in M applies to N–P. bsr, branchiostegal ray; cb, ceratobranchial arches; ch, ceratohyal; cl, cleithrum; d, dentary; e, ear; ep, ethmoid plate; m, maxilla; op, operculum; s, somites; t, tooth; tb, trabecule.

Wholemount in situ hybridization showed that *col27a1a* is expressed in the notochord (Fig. 2A–I, K, L). Expression was dynamic, beginning during late epiboly (Fig. 2A-B) and spreading to the anterior three-quarters of the notochord by 24 hpf (Fig. 2I), and then throughout the notochord by 30 hpf (data not shown). Subsequently, notochordal expression became restricted to the distal tip of the tail by 48 hpf (Fig. 2L) and was no longer detectable by 72 hpf. *col27a1a* transcript was detected throughout the floor plate and hypochord as well at 24 hpf (Fig. 2K). This pattern in the notochord and surrounding tissues is similar to that of type II collagen [17]. *col27a1a* was also expressed in forming head cartilages (Fig. 2L–P) and in the first forming tooth (Fig. 2M), similar to the reported expression domain of *Col27a1* in mouse [8]–[9]. By 5 dpf, when mineralization of craniofacial cartilage elements has already commenced, *col27a1a* expression was restricted to the periphery of many of these elements (Fig. 2N, P).

**Figure 3:**
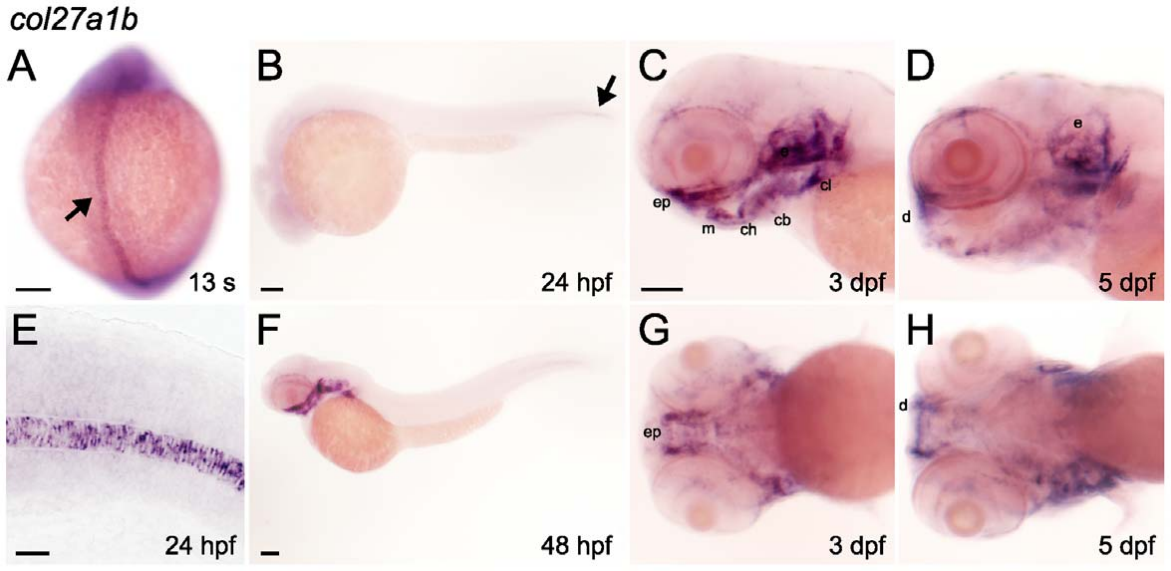
*col27a1b* is expressed in notochord and cartilage. *col27a1b* was expressed throughout the notochord at 13 somites (A) but by 24 hpf (B) was restricted to the distal tip of the tail (black arrow). (E) *col27a1b* expression in the notochord was restricted to the notochord itself and was not present in surrounding structures. (F) Expression of *col27a1b* in cartilage was observed beginning at 48hpf. Cartilage expression was also seen at 3 dpf (C, G) and 5 dpf (D, H), but it was diffuse and individual cartilage structures were not easily distinguished. Scale bar in E represents 20 µm. Scale bars in remaining images represent 100 µm. Scale bar in C applies to D, G, and H. cb, ceratobranchial arches; ch, ceratohyal; cl, cleithrum; d, dentary; e, ear; ep, ethmoid plate; m, maxilla.

*col27a1b* was expressed in a similar but distinct pattern to that of *col27a1a* (Fig. 3). Weak expression was observed at the 6 somite stage in the notochord (data not shown). At 13 somites it was expressed throughout the notochord (Fig. 3A), but by 24 hpf, expression was restricted to the distal tip of the notochord (Fig. 3B, E). *col27a1b* was also expressed in head cartilages by 48 hpf (Fig. 3F) and expression was observed through the latest time point analyzed (5 dpf) (Fig. 3C,D,G,H).

### *col27a1a* Is Required for Post-Embryonic Axial Skeletogenesis

To test roles for type XXVII collagen in notochord development and formation of craniofacial cartilages, we used morpholino antisense oligonucleotides to knock-down synthesis of normal type XXVII collagen protein. As an initial test of *col27a1a* function, we designed a splice-blocking morpholino (SBMO) [18]–[19] to target the splice donor site of intron 58. *col27a1a* contains 61 exons, most of which are in frame such that potential alternative splicing of an SBMO-targeted transcript could leave the open reading frame intact. We chose exon 58 for targeting since a frame-shift and premature stop codon in exon 59 would result, in the unlikely event of a mis-spliced transcript joining exons 57 and 59.

**Figure 4:**
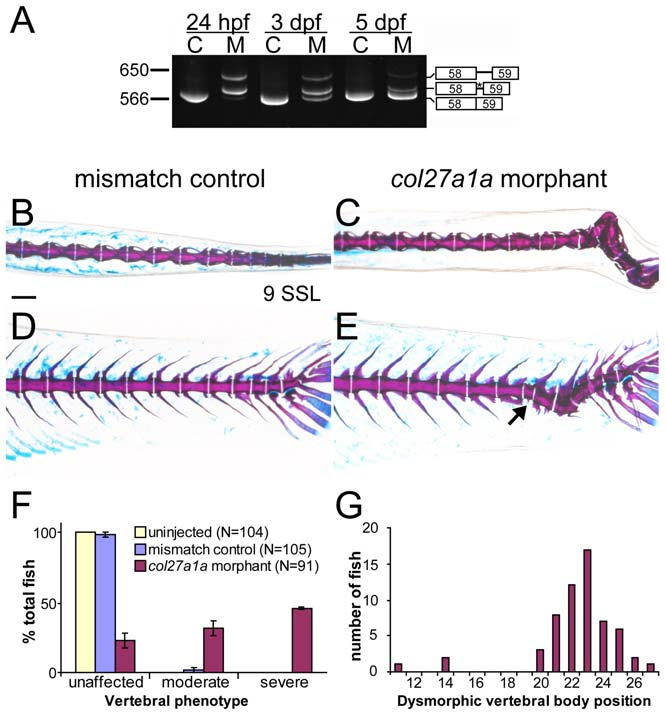
*col27a1a* morphants develop scoliosis and dysmorphic vertebrae. (A) Embryos injected with a SBMO targeting the exon 58 splice donor site of *col27a1a* showed abnormal splicing. Two abnormal splice forms including portions of intron 58 were generated. One splice form was predicted to generate a premature stop codon (asterisk). C, control; M, morphant. Dorsal and lateral views of *col27a1a* morphants (C, E) and mismatch control siblings (B, D) at ∼8.1–9.0 SSL (21 dpf) showed formation of scoliotic curves in the morphant vertebral column. Morphants were injected with a combined dose of 5 ng *col27a1a* SBMO+1 ng *col27a1a* TBMO. (F) By ∼8.1–9.0 SSL, 78% of *col27a1a* morphants had developed either moderate or severe vertebral defects. The mean from 3 clutches±standard error is shown. (E) Dysmorphic vertebrae without hemal or neural spines were also observed in the morphant vertebral column (black arrow). (G) These narrow, abnormal vertebrae were preferentially localized near the distal end of the tail. Vertebrae were counted beginning immediately distal to the 4 modified vertebrae of the Weberian apparatus. Scale bar in A represents 200 µm and applies to B–D.

Morpholino dosages for SBMO and other morpholinos (below) were determined according to standard procedures using 5 base pair (bp) mismatch morpholinos (mmMO) [20]. RT-PCR analysis of the morpholino-targeted region showed abnormal splicing of *col27a1a* in embryos injected with *col27a1a* SBMO and normal splicing in uninjected control embryos (Fig. 4A). Sequencing of PCR amplicons revealed that one abnormal splice product included 23 bp of intron 58, which introduced a premature stop codon and would likely be subjected to nonsense-mediated RNA decay. A second variant included 57 bp of intron 58, causing it to be in-frame and likely resulting in an abnormal translated protein (Fig. 4A). No abnormal splicing of the *col27a1b* transcript was observed, indicating that the morpholino is specific for *col27a1a* (data not shown). We also designed a translation-blocking morpholino (TBMO) targeting the translation start site of *col27a1a*. The results described below were observed with both the *col27a1a* SBMO and TBMO individually, but to enhance *col27a1a* knockdown and to prevent off-target effects, experiments shown were performed using a combination of *col27a1a* SBMO (5 ng) and *col27a1a* TBMO (1 ng) with the same combined dosages of the corresponding mismatch morpholinos as a control. The results described below could not be rescued by co-injection with a morpholino targeting p53, indicating that they are not off-target effects due to p53 activation [21].

Embryos injected with *col27a1a* MOs showed no overt phenotype in notochord, cartilage, or somites prior to 5 dpf (data not shown). By contrast, when morphants developed to post-embryonic stages (∼8.1–9.0 SSL, 21 dpf) we frequently observed curvature of the fully mineralized vertebral column, as well as abnormal morphologies of individual vertebrae (Fig. 4C,E). Vertebral column defects generally consisted of lateral curvature though downward curvature also was seen occasionally. We classified phenotypes as either unaffected, moderately defective (vertebral segmentation defects and malformed vertebrae with minimal lateral curvature), or severely defective (lateral curvature of vertebrae usually accompanied by highly malformed vertebrae and vertebral segmentation defects). We found high frequencies of the latter classes in morpholino-treated individuals but not in mismatch controls or uninjected fish (Fig. 4F). We also observed formation of dysmorphic vertebrae lacking hemal and neural spines (Fig. 4E). These abnormal vertebrae were most likely to arise slightly anterior to the distal tail, where severe spinal curves formed (Fig. 4G). Insertion of additional vertebrae in such a defined region suggests a specific alteration in patterning.

### *col27a1a* Is Essential for Notochord Morphogenesis and Promotes Early Vertebral Development and Mineralization

**Figure 5:**
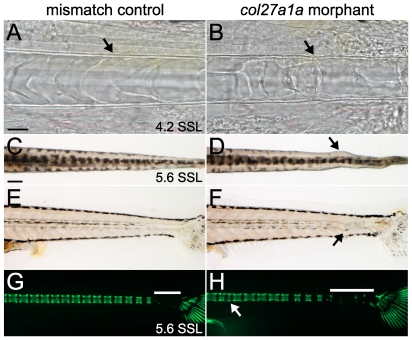
*col27a1a* is required for notochord development. Brightfield analysis of morphant (B) and mismatch control (A) notochords at ∼4.2 SSL (8 dpf) showed buckling of the notochord sheath. Dorsal (D) and lateral (F) views of *col27a1a* morphants at ∼5.6 SSL (14 dpf) show formation of notochord curves at the distal end of the tail. (C, E, G) Mismatch control siblings. (H) Calcein staining to visualize mineral showed abnormal vertebrae (white arrow) and decreased mineralization (white bar indicates unmineralized region). Scale bar in A represents 20 µm and applies to B. Scale bar in C represents 200 µm and applies to D–H.

We were surprised to observe post-embryonic vertebral defects since morpholino effects on RNA splicing and protein abundance typically last for <7 days (Fig. 4A) [18]. Because *col27a1a* is expressed in the notochord and surrounding structures when morpholinos are effective, we hypothesized that knockdown of *col27a1a* in these cells was responsible for the observed changes in vertebral formation and patterning. We therefore examined the notochord at earlier stages and at higher resolution. There was buckling of the notochord sheath as early as ∼4.2 SSL (8 dpf; Fig. 5B), prior to the onset of curve formation, which was detectable only in some morphants by ∼4.2–4.5 SSL (8–10 dpf), but was more typically apparent during later development (Fig. 5D, F).

**Figure 6:**
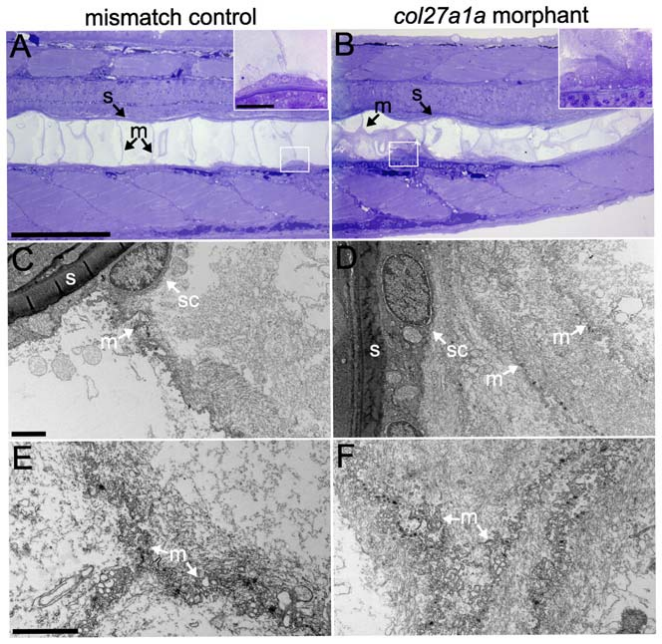
Protein and cellular accumulation in the *col27a1a* morphant notochord. Semithin sections (300 nm) of mismatch control (A) and *col27a1a* morphant (B) notochord were stained with Richardson's stain for proteins at ∼4.5 SSL (10 dpf). The morphant notochord showed abnormal cellular clumping along the notochord sheath (white box and inset) and increased staining along membranes between notochord cells. (C–F) TEM analysis indicated that this staining was due to increased protein accumulation along membranes separating notochord cells. Scale bar in A represents 200 µm and applies to B. Scale bar in A inset represents 20 µm and applies to B inset. Scale bars in C and E represent 2 µm and apply to D and F, respectively. s, notochord sheath; sc, sheath cell; m, membrane.

To further assess the nature of notochord defects in morphant embryos, we analyzed notochord structure in *col27a1a* morphants by transmission electron microscopy at the earliest stage in which we could detect irregularities by light microscopy (∼4.5 SSL). In fish injected with a control mismatch morpholino, a row of large, vacuolated cells surrounded by a thick collagenous sheath was observed. A row of cells, referred to here as “sheath cells,” lined the inner surface of the sheath (Fig. 6A,C). The morphant notochord, however, displayed an accumulation of material within curved regions as well as cells clumped along the inner surface of the sheath (Fig. 6B). We further observed significant accumulation of material, likely to be mislocalized protein, along cell membranes within the notochord curves (Fig. 6B,D,F). These data suggest that notochord curve formation is accompanied by abnormal protein deposition.

Since fibrillar collagens can serve as scaffolds for proteoglycans and for mineral deposition, we hypothesized that disruption of notochord structure by *col27a1a* knockdown could influence subsequent vertebral form through effects on vertebral mineralization. We tested this hypothesis by staining *col27a1a* morphants with calcein at early stages of vertebral mineralization (∼5–5.5 SSL, 12 dpf). There was a significantly reduced number of mineralized vertebrae in morphants as compared to sibling mismatch controls (Fig. 5G, H; *F*_2,334_ = 19.2, *P*<0.0001; mean±SE mineralized vertebrae for morphants and mismatch control, respectively: 24.6±0.30; 26.7±0.30).

We considered the possibility that delays in vertebral mineralization might have resulted from a general developmental retardation in morphant individuals, although morphants and mismatch controls did not differ noticeably in overall developmental stage. We determined the numbers of mineralized vertebrae in sibling morphants and mismatch controls at ∼3.6–4.0 SSL (6 dpf), before the earliest onset of vertebral curve formation. We then measured the actual standard length of each individual, which is a proxy for developmental stage in post-embryonic larvae [11], and we tested if differences in mineralized vertebral number remained after controlling for differences in standard length. These analyses revealed that even after controlling for individual variation in standard length (*F*_1,212_ = 124.7, *P*<0.0001), there remained a significant reduction in the number of mineralized vertebrae in morphants compared to mismatch controls and uninjected siblings (*F*_2,212_ = 18.7, *P*<0.005; Tukey-Kramer post hoc comparison, *P*<0.05; mean±SE mineralized vertebrae for morphants and mismatch control, respectively: 3.4±0.13, 3.8±0.13). Finally, we did not detect an overall developmental delay in any of several other diagnostic traits at these stages [11]. Together these analyses indicate a role for *col27a1a* in promoting the normal rate of vertebral mineralization and support a model in which early embryonic defects in a template for mineralization result in post-embryonic defects in vertebral form.

### *col27a1a* and *col27a1b* Function within the Same Genetic Pathway in Zebrafish Notochord

**Figure 7:**
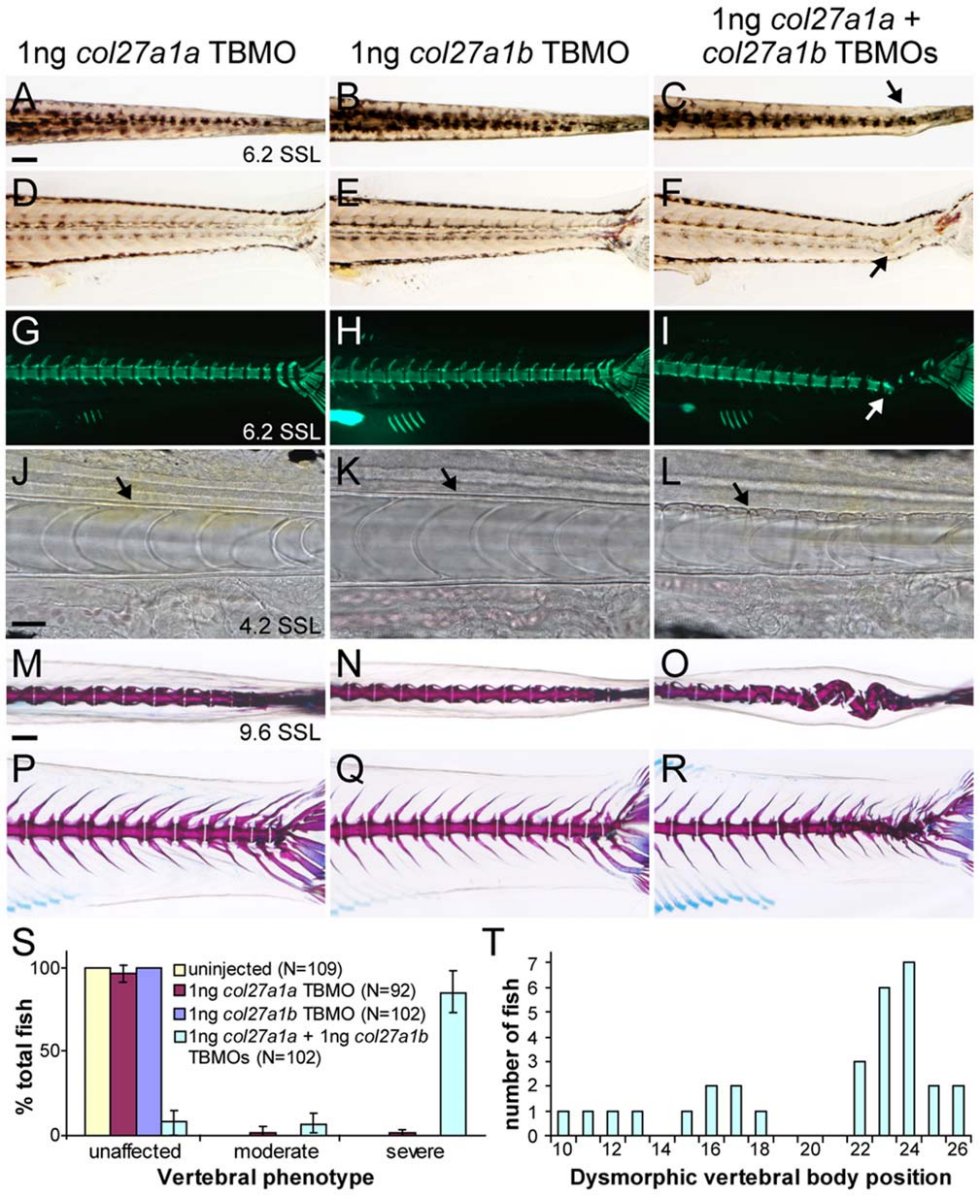
*col27a1a* and *col27a1b* interact genetically in the notochord and axial skeleton. Injection of *col27a1a* TBMO (A, D, G) and *col27a1b* TBMO (B, E, H) at low dosage did not result in a phenotype as seen at ∼6.2 SSL (14 dpf). (C, F) Simultaneous injection of *col27a1a* and *col27a1b* TBMOs at low dosage, however, resulted in notochord curves (black and white arrows). (I) Calcein staining to visualize mineral showed abnormal mineralization in double morphants. Brightfield analysis of *col27a1a* (J), *col27a1b* (K) and *col27a1a* and *col27a1b* double morphant (L) notochords at ∼4.2 SSL (8 dpf) showed severe buckling of the notochord sheath (black arrow) in double morphants. Dorsal and lateral views of *col27a1a* (M, P), col27a1b (N, Q) and double morphants (O, R) at ∼9.6–10 SSL (21 dpf) showed formation of scoliotic curves in the vertebral column of double morphants. (S) By ∼9.6–10 SSL, 92% of *col27a1a* and *col27a1b* double morphants had developed either moderate or severe vertebral defects, with the majority being very severe. The mean from 3 clutches±standard error is shown. (T) Dysmorphic vertebrae without hemal or neural spines were also observed in double morphants. These additional dysmorphic vertebrae were spread throughout the vertebral column but preferentially localized to a region near the distal end of the tail. Scale bars in A and M represent 200 µm and apply to B–I and N–R, respectively. Scale bar in J represents 20 µm and applies to K and L.

In contrast to *col27a1a*, targeting of *col27a1b* with a translation-blocking morpholino (TBMO) or a splice-blocking morpholino (SBMO) did not yield patent defects in vertebral morphology, despite the induction of abnormal splicing by the latter (data not shown). The inefficacy of *col27a1b* morpholino knockdown could have resulted from functional redundancy between *col27a1a* and *col27a1b* paralogues. To test the hypothesis that *col27a1a* compensates for knockdown of *col27a1b*, and to determine if these loci function in the same molecular pathway, we injected zebrafish embryos simultaneously with *col27a1a* and *col27a1b* TBMOs at dosages (1 ng) that did not individually cause morphological defects. Similar to *col27a1a* morphants, *col27a1a* and *col27a1b* double morphants developed notochord curves and mineralized vertebrae within these curves, resulting in scoliosis (Fig. 7). As in *col27a1a* single morphants, the earliest sign of curve formation was buckling of the notochord sheath (Fig. 7J–L). At ∼9.6–10 SSL (21 dpf), vertebrae at the distal tip of the tail were severely deformed, and dysmorphic vertebrae lacking hemal and neural spines were also observed (Fig. 7O,R). Severe vertebral defects were observed in 85% of double morphants (Fig. 7S), and due to the severity of the spinal curves, it was difficult to determine if additional dysmorphic vertebrae were present since individual vertebrae could not be easily distinguished within the curved regions. For those fish in which localization could be determined, the distribution of these abnormal vertebrae appeared more widespread than in *col27a1a* single morphants (Fig. 7T).

We also assayed *col27a1a* and *col27a1b* double morphants for mineralization defects. At ∼5.2–5.7 SSL (12 dpf), double morphants showed a statistically significant delay in vertebral mineralization (*F*_3,336_ = 22.0, *P*<0.0001; mean±SE mineralized vertebrae: *col27a1a*, 27.4±0.22; *col27a1b*, 29.0±0.22; *col27a1a* and *col27a1b*, 26.6±0.22). Uninjected controls and *col27a1a* morphants injected with a sub-effective dose of *col27a1a* TBMO (1 ng) showed no significant difference in mineralization whereas mineralization in *col27a1b* morphants injected with 1 ng *col27a1b* TBMO was, surprisingly, slightly accelerated relative to uninjected controls. To confirm that delayed mineralization in the double morphants was not linked to a developmental delay, additional fish were scored for both vertebral mineralization and standard length at ∼3.7–4.1 SSL as above. This analysis confirmed that even after accounting for variation in standard length (*F*_1,176_ = 88.4, *P*<0.0001) there was a significant reduction in vertebral mineralization in double morphants, as compared to single morphants (*F*_2,176_ = 33.4, *P*<0.0001; Tukey Kramer post hoc comparison, *P*<0.05; mean±SE mineralized vertebrae: *col27a1a*, 6.0±0.23; *col27a1b*, 7.9±0.24; *col27a1a* and *col27a1b*, 5.2±0.22). These data show that *col27a1a* and *col27a1b* work in concert in the developing notochord and both play an important role in development of the zebrafish axial skeleton.

## Discussion

We identified two genes for type XXVII collagen in zebrafish and demonstrated a critical role for their protein products in notochord development and post-embryonic axial growth. Knockdown of *col27a1a* singly or concurrently with *col27a1b* results in the development of notochord curves and a scoliotic adult phenotype with formation of dysmorphic vertebrae. The earliest signs of a notochord defect include buckling of the notochord sheath, abnormal accumulation of protein in the notochord, and a delay in vertebral mineralization.

As shown previously for mouse *Col27a1*, zebrafish *col27a1a* and *col27a1b* were expressed in cartilage [8]. Studies of mouse also showed expression of *Col27a1* in presumptive vertebrae but notochord expression was not analyzed. Here we have shown expression of *col27a1a* and *col27a1b* in the notochord at early stages. To address the function of type XXVII collagen, we used a morpholino knockdown strategy. While rescue of the morpholino phenotype was not possible owing to the large sizes of these collagen transcripts [20], the specificity of our morpholino effects are supported by the use of low combined doses that did not individually cause defects, and by the failure of p53 knockdown to rescue the observed phenotypes.

The structure of the notochord is characterized by a row of large, vacuolated cells that exert pressure on a thick collagenous sheath to form a type of hydrostatic skeleton in the embryo and young larvae [22]. Buckling of the notochord sheath in *col27a1a* morphants and *col27a1a*/*col27a1b* double morphants suggests that knockdown of *col27a1a* and *col27a1b* results in a weak sheath that is unable to withstand the force generated by the cells within the notochord. Distinct scoliotic curves are formed preferentially at the distal tip of the notochord, and excess protein is deposited at sites of curve formation. The vertebrae mineralize around these abnormal protein deposits, which may explain the severe structural alterations we observed. Previous work demonstrated that type XXVII collagen accumulates at the site of cartilage to bone transition [23], consistent with the role in mineralization postulated here.

We have shown that embryos deficient in *col27a1a* mineralize vertebrae more slowly than control siblings, indicating a role for this locus in promoting mineralization during normal development. Our analyses showed a similar delay of mineralization—as well as notochord curvature and vertebral malformations—in double morphants for *col27a1a* and *col27a1b*, after combined injection of low doses of translation-blocking morpholinos that did not, individually, yield overt morphological defects. By contrast, mineralization was accelerated in embryos injected with *col27a1b* translation-blocking morpholino alone. These alternative outcomes may reflect biochemical interactions between the protein products of the *col27a1a* and *col27a1b* loci. The mature collagen molecule comprises three collagen chains, and we expect that the protein products of *col27a1a* and *col27a1b* can form either homotrimers or heterotrimers having different consequences for mineralization rate. Thus, differences in the absolute abundance of the two protein products, the ratios of homotrimers to heterotrimers, or the ratios of different heterotrimer species all could affect mineralization outcome. We speculate that lower expression of *col27a1b* compared to *col27a1a*, the absence of morphological defects in *col27a1b* single morphants, and the presence of a novel third interruption in the triple helix in the inferred protein product of *col27a1b* may together reflect its incipient non-functionalization, following duplication of an ancestral *col27a1* locus. Given the likelihood of *col27a1a* and *col27a1b* interactions, the accelerated mineralization upon knock-down of *col27a1b* alone may indicate that its gene product now interferes weakly with the mineralization process, the precise timing of which presumably depends on a specific balance of *col27a1a* and *col27a1b* homotrimers and heterotrimers.

Unlike most reported morphant phenotypes, the *col27a1a* and *col27a1b* morphant phenotype is not clearly apparent until the morpholino is no longer active. This suggests that *col27a1a* and *col27a1b* are required during early development and are critical for formation of later structures. Similar early embryonic requirements for the development of later adult form have been demonstrated in other systems [24]–[25]. Deposition of type XXVII collagen in the matrix may serve as a signal for synthesis of other proteins critical for later stages of notochord development. Alternatively, early depletion of *col27a1a* and *col27a1b* may alter early notochord structure at a level that we have not been able to detect, changing later protein interactions and affecting notochord development. We favor this hypothesis since early buckling of the notochord suggests a mechanical weakening of the notochord sheath. We speculate that when pressures applied by rapid growth or movement during the larval period are applied to the weakened sheath, it buckles, causing the notochord to curve.

A guppy model of scoliosis has been characterized and many zebrafish notochord mutants have also been identified, indicating that scoliosis is not restricted to humans or other ambulatory animals [26]–[27]. The *col27a1a* and *col27a1b* morphant appears to most closely resemble a group of zebrafish notochord mutants characterized by a folded notochord [28]. This group includes mutants with mutations in *col8a1* (type VIII collagen) [29], *knypek* (a heparan sulfate proteoglycan) [30], and *trilobite*, which encodes a transmembrane protein involved in canonical Wnt/β-catenin signaling [31]. Mutations in *trilobite* and *knypek* have been linked to defects in convergence and extension movements during gastrulation while *col8a1* mutants show disruption of the fibrillar layer in the notochord sheath and protein aggregation in notochord cells. In contrast to the *col27a1a* and *col27a1b* morphants, the *col8a1* mutant shows notochord defects by 24 hpf and the notochord straightens by 3 dpf (pleiotropic defects would prevent analysis of a late phenotype) [28]. This implies different requirements for notochord sheath structure during different developmental stages and suggests that there remains much to learn about the notochord sheath and its roles across stages. Inhibition or knockdown of lysyl oxidases, enzymes that cross-link collagens within the extracellular matrix, also result in an undulating notochord phenotype, confirming an important role for collagens and their related enzymes in notochord development and structure [32]–[34]. Moreover, lysyl oxidases are copper-dependent enzymes, and mutation of a copper transporting ATPase in a background of copper-deficiency results in post-embryonic vertebral defects similar to those described here for *col27a1a* and *col27a1b* morphants [35].

As nearly all fibrillar collagens are linked to human disease, we suspect that mutations in type XXVII collagen may also lead to disease. We previously sequenced *COL27A1* in patients with various chondrodysplasias, since mRNA and protein for type XXVII collagen were identified in cartilage [8], [23]. However, we were unable to identify cartilage or other craniofacial defects in *col27a1a* and *col27a1b* zebrafish morphants at early or late stages, and our results suggest that the notochord is a key site of type XXVII collagen function. Analysis of the morphant phenotype as described in this study showed that zebrafish deficient in type XXVII collagen develop abnormal, dysmorphic vertebrae, indicating that the phenotype most closely resembles forms of congenital scoliosis. The genetic components of congenital scoliosis are largely unknown though mutations in the gene for Notch ligand delta-like 3 have been identified in a subset of individuals with vertebral segmentation defects [36]–[37]. There are many disorders characterized by congenital vertebral defects and mutations in *COL27A1* may play a role in the etiology of one or more of these disorders.
